# Supplementary material for: HEHR: Homing Endonuclease-Mediated Homologous Recombination for Efficient Adenovirus Genome Engineering
Source: Genes (Basel). 2022 Nov 16;13(11):2129. doi: 10.3390/genes13112129 (PMC9690551; doi:10.3390/genes13112129)
Supplement: Supplementary file 1 [file genes-13-02129-s001.zip › genes-1983687-supplementary.pdf]

Table S1. Non-cutters scanning of human adenovirus types

S1-1. Species A

**HAd types**	AbsI	AsiSI	BanIII	Bsa29I	BseCI	BshVI	BspDI	BspXI	BstSNI	Bsu15I	BsuTUI	ClaI	CpoI	CspI	Eco105I	FseI	I-CeuI	I-PpoI	I-SceI	MreI	PI-PspI		PI-SceI	RgaI	RigI	RsrII	Rsr2I	SbfI	SdaI	SfaAI	SfiI	SgfI	SgrDI	SmiI	SnaBI	SrfI	Sse8387I	SwaI
12	X		X	X	X	X	X	X	X	X	X	X	X	X	X	X	X	X	X		X		X		X	X	X	X	X		X			X	X	X	X	X
18	X								X				X	X	X	X	X	X	X	X	X		X		X	X	X						X	X	X			X
31	X	X											X	X			X	X	X		X		X	X		X	X	X	X	X	X	X					X	
61	X	X	X	X	X	X	X	X		X	X	X	X	X		X	X	X	X		X		X	X	X	X	X	X	X	X	X	X					X	

S1-2. Species B

**HAd types**	AbsI	AsiSI	CciNI	CpoI	CspI	FseI	I-CeuI	I-PpoI	I-SceI	MauBI	MluI	MssI	NotI	PI-PspI	PI-SceI	PmeI	RgaI	RigI	RsrII	Rsr2I	SbfI		SdaI	SfaAI	SfiI	SgfI	SgrDI	SmiI	SrfI	Sse8387I	SwaI
3	X	X				X	X	X	X		X	X		X	X	X	X	X			X		X	X	X	X	X	X	X	X	X
7	X	X		X	X	X	X	X	X			X		X	X	X	X	X	X	X	X		X	X	X	X	X	X	X	X	X
11	X	X	X			X	X	X	X	X		X	X	X	X	X	X	X			X		X	X	X	X		X	X	X	X
14	X	X	X			X	X	X	X	X		X	X	X	X	X	X	X			X		X	X	X	X		X	X	X	X
16	X	X				X	X	X	X		X			X	X		X	X			X		X	X		X	X	X	X	X	X
21	X	X	X			X	X	X	X			X	X	X	X	X	X	X						X		X	X	X	X		X
34	X	X	X			X	X	X	X	X		X	X	X	X	X	X	X			X		X	X	X	X		X	X	X	X
35	X	X	X			X	X	X	X	X		X	X	X	X	X	X	X			X		X	X	X	X		X	X	X	X
50	X	X	X			X	X	X	X			X	X	X	X	X	X	X			X		X	X		X	X	X	X	X	X
55	X	X	X			X	X	X	X	X		X	X	X	X	X	X	X			X		X	X	X	X		X	X	X	X
66	X	X					X	X	X		X	X		X	X	X	X				X		X	X	X	X	X	X	X	X	X
68	X	X				X	X	X	X		X	X		X	X	X	X	X			X		X	X	X	X	X	X	X	X	X

S1-3. Species C

**HAd types**	AbsI	AsiSI	AsuII	BarI	Bpu14I	Bsp119I	BspT104I	BstBI	BstSNI	Csp45I	Eco105I	FauNDI	I-CeuI	I-PpoI	I-SceI	NdeI	NspV	PacI	PI-PspI	PI-SceI	PsrI		RgaI	SfaAI	SfuI	SgfI	SgrDI	SmiI	SnaBI	SwaI
1	X	X	X	X	X	X	X	X	X	X	X	X	X	X	X	X	X	X	X	X	X		X	X	X	X	X		X	
2	X								X		X		X	X	X				X	X							X		X	
5	X		X		X	X	X	X		X			X	X	X		X	X	X	X	X				X		X	X		X
6	X	X							X		X		X	X	X				X	X			X	X		X	X		X	

S1-4. Species D

HAd types	AclI	AhlI	AseI	AsiSi	BarI	BcuI	BssNAI	Bst1107I	BstHPI	BstSNI	BstZ17I	Eco105I	HpaI	I-CeuI	I-PpoI	I-SceI	KspAI	MluI	MssI	PacI	PI-PspI	PI-SceI	PmeI	Psp1406I	PshBI	RgaI	SfaAI	SgfI	SmiI	SnaBI	SpeI	SrfI	SwaI	VspI
8				X			X	X		X	X	X		X	X	X			X		X	X	X			X	X	X	X	X			X	
9		X		X		X				X		X		X	X	X			X		X	X	X			X	X	X	X	X	X		X	
10		X		X		X				X		X		X	X	X			X		X	X	X			X	X	X	X	X	X		X	
13		X		X		X	X	X	X	X	X	X	X	X	X	X	X		X	X	X	X	X			X	X	X	X	X	X		X	
15		X	X	X		X				X		X		X	X	X			X	X	X	X	X		X	X	X	X	X	X	X		X	X
17				X						X		X		X	X	X			X	X	X	X	X			X	X	X	X	X			X	
19	X	X				X				X		X		X	X	X			X		X	X	X	X		X	X	X	X	X	X	X	X	
20				X										X	X	X			X		X	X	X			X	X	X	X				X	
22				X					X	X		X	X	X	X	X	X		X	X	X	X	X			X	X	X		X				
23				X						X		X		X	X	X			X	X	X	X	X			X	X	X	X	X			X	
24				X			X	X			X			X	X	X			X		X	X	X			X	X	X	X				X	
25		X		X		X	X	X	X	X	X	X	X	X	X	X	X		X	X	X	X	X			X	X	X	X	X	X	X	X	
26		X		X		X	X	X	X	X	X	X	X	X	X	X	X		X	X	X	X	X			X	X	X	X	X	X		X	
27		X		X		X				X		X		X	X	X			X	X	X	X	X			X	X	X	X	X	X		X	
28	X	X	X	X		X				X		X		X	X	X			X	X	X	X	X	X	X	X	X	X	X	X	X		X	X
29		X		X		X	X	X	X	X	X	X	X	X	X	X	X		X	X	X	X	X			X	X	X	X	X	X		X	
30				X			X	X	X	X	X	X	X	X	X	X	X		X	X	X	X	X			X	X	X		X				
32				X			X	X		X	X	X		X	X	X			X	X	X	X	X			X	X	X		X		X		
33				X						X		X		X	X	X			X	X	X	X	X			X	X	X	X	X			X	
36				X						X		X		X	X	X		X	X		X	X	X			X	X	X	X	X			X	
37		X		X		X	X	X	X	X	X	X	X	X	X	X	X	X	X	X	X	X	X			X	X	X		X	X			
38				X										X	X	X			X	X	X	X	X			X	X	X	X			X	X	
39	X			X						X		X		X	X	X			X	X	X	X	X	X		X	X	X	X	X			X	
42		X		X		X				X		X		X	X	X			X	X	X	X	X			X	X	X	X	X	X		X	
43	X	X		X		X	X	X	X	X	X	X	X	X	X	X	X		X	X	X	X	X	X		X	X	X	X	X	X		X	
44				X			X	X			X			X	X	X			X	X	X	X	X			X	X	X	X				X	
45				X			X	X	X	X	X	X	X	X	X	X	X		X		X	X	X			X	X	X		X				
46				X			X	X		X	X	X		X	X	X			X		X	X	X			X	X	X	X	X			X	
47		X		X		X	X	X		X	X	X		X	X	X			X	X	X	X	X			X	X	X	X	X	X		X	
48				X										X	X	X			X	X	X	X	X			X	X	X	X			X	X	
49	X			X			X	X	X	X	X	X	X	X	X	X	X		X	X	X	X	X	X		X	X	X		X				
51	X	X		X		X				X		X		X	X	X			X	X	X	X	X	X		X	X	X	X	X	X		X	
53		X		X		X	X	X	X	X	X	X	X	X	X	X	X		X	X	X	X	X			X	X	X	X	X	X		X	
54				X			X	X		X	X	X		X	X	X			X		X	X	X			X	X	X	X	X			X	
56		X		X		X	X	X		X	X	X		X	X	X			X		X	X	X			X	X	X	X	X	X		X	
58	X	X		X		X	X	X		X	X	X		X	X	X		X	X	X	X	X	X	X		X	X	X	X	X	X		X	

S1-4. Species D (continued)

HAd types	AclI	AhlI	AseI	AsiSi	BarI	BcuI	BssNAI	Bst1107I	BstHPI	BstSNI	BstZ17I	Eco105I	HpaI	I-CeuI	I-PpoI	I-SceI	KspAI	MluI	MssI	PacI	PI-PspI	PI-SceI	PmeI	Psp1406I	PshBI	RgaI	SfaAI	SgfI	SmiI	SnaBI	SpeI	SrfI	SwaI	VspI
63		X		X		X	X	X	X	X	X	X	X	X	X	X	X		X	X	X	X	X			X	X	X	X	X	X		X	
64				X	X				X	X		X	X	X	X	X	X	X	X	X	X	X	X			X	X	X		X				
65	X			X			X	X		X	X	X		X	X	X		X	X		X	X	X	X		X	X	X	X	X			X	
67	X	X		X		X	X	X	X	X	X	X	X	X	X	X	X			X	X	X		X		X	X	X		X	X			
69				X			X	X			X			X	X	X			X	X	X	X	X			X	X	X	X				X	
70	X	X		X		X								X	X	X			X	X	X	X	X	X		X	X	X	X		X		X	
71				X			X	X	X	X	X	X	X	X	X	X	X		X	X	X	X	X			X	X	X	X	X		X	X	
73				X						X				X	X	X			X	X	X	X	X			X	X	X	X	X			X	
74				X						X		X	X	X	X	X	X		X	X	X	X	X			X	X	X	X	X			X	
75	X	X		X		X				X		X		X	X	X			X	X	X	X	X	X		X	X	X	X	X	X		X	
81				X					X	X		X	X	X	X	X	X		X	X	X	X	X			X	X	X		X		X		
86	X	X		X		X	X	X		X	X	X		X	X	X			X	X	X	X	X	X		X	X	X	X	X	X		X	

S1-5. Species E, F and G

**Species**	**HAd types**	AbsI	AhlI	AscI	AsiSI	BcuI	CciNI	FseI	I-CeuI	I-PpoI	I-SceI	MauBI	MluI	MssI	NotI	PacI	PalAI	PI-PspI	PI-SceI	PmeI	RgaI	RigI	SanDI	SbfI	SdaI	SfaAI	SgfI	SgrDI	SgsI	SmiI	SpeI	SrfI	Sse8387I	SwaI
E	4	X	X		X	X				X						X		X	X		X					X	X			X	X	X		X
F	40	X			X				X	X	X	X						X	X		X		X	X	X	X	X						X	
	41	X		X			X		X	X	X	X	X	X	X		X	X	X	X				X	X			X	X				X	
G	52	X						X	X	X	X					X		X	X			X		X	X			X		X			X	X

Figure S1. Plasmid map and DNA sequence of the plasmids described in this protocol

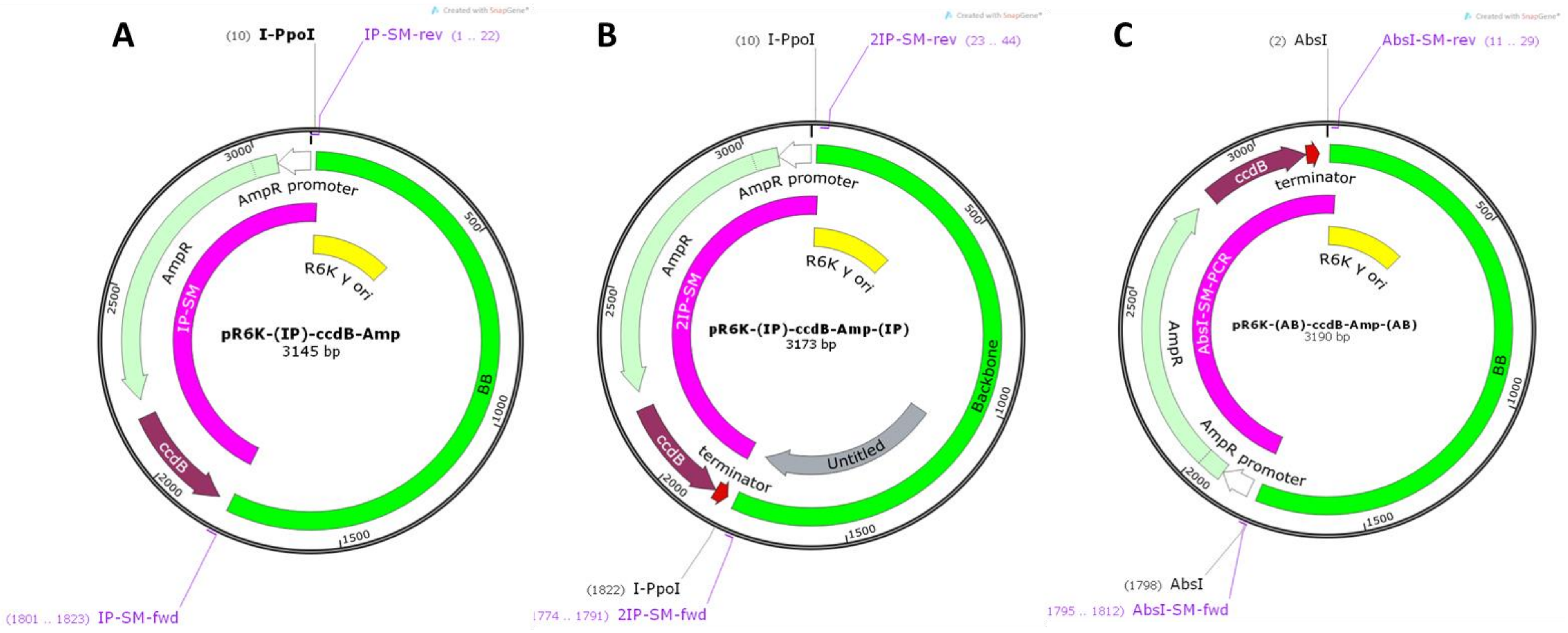

A. pR6K-(IP)-ccdB-Amp serves as PCR template to amplify the ccdB-Amp cassette. The R6K backbone is used to avoid background resistance in recombineering. Primers IP-SM-fwd (**bold and underlined**, see the sequence below) and IP-SM-rev (reverse to the region ***bold and italic***, see the sequence below) are used to amplify the selection marker cassette.

B. pR6K-(IP)-ccdB-Amp-(IP) serves as PCR template to amplify the ccdB-Amp cassette. The R6K backbone is used to avoid background resistance in recombineering. Primers 2IP-SM-fwd (**<u>bold and underlined</u>**, see the sequence below) and 2IP-SM-rev (reverse to the region ***bold and italic***, see the sequence below) are used to amplify the selection marker cassette.

C. pR6K-(AB)-ccdB-Amp-(AB) serves as PCR template to amplify the ccdB-Amp cassette. The R6K backbone is used to avoid background resistance in recombineering. Primers AB-SM-fwd (**<u>bold and underlined</u>**, see the sequence below) and AB-SM-rev (reverse to the region ***bold and italic***, see the sequence below) are used to amplify the selection marker cassette.

Sequence:

A. pR6K-(IP)-ccdB-Amp

GCTACCTTAAGAGAGTGTCAGCCGTTAAGTGTTCCTGTGTCACTCAAAATTGC***TTTGAGAGGCTCTAAGGGCTTC***TCAGTGCGTTACATCCCTGGC
TTGTTGTCCACAACCGTTAAACCTTAAAAGCTTTAAAAGCCTTATATATTCTTTTTTTTCTTATAAAACTTAAAACCTTAGAGGCTATTTAAGTTGC
TGATTTATATTAATTTTATTGTTCAAACATGAGAGCTTAGTACGTGAAACATGAGAGCTTAGTACGTTAGCCATGAGAGCTTAGTACGTTAGCCA
TGAGGGTTTAGTTCGTTAAACATGAGAGCTTAGTACGTTAAACATGAGAGCTTAGTACGTGAAACATGAGAGCTTAGTACGTACTATCAACAGG
TTGAACTGCTGATCTTCAGATCCTCTACGCCGGACGCATCGTGGCCGTTTTCCGCTGCATAACCCTGCTTCGGGGTCATTATAGCGATTTTTTCGG
TATATCCATCCTTTTTCGCACGATATACAGGATTTTGCCAAAGGGTTCGTGTAGACTTTCCTTGGTGTATCCAACGGCGTCAGCCGGGCAGGATA
GGTGAAGTAGGCCCACCCGCGAGCGGGTGTTCCTTCTTCACTGTCCCTTATTCGCACCTTAATTAATTCACTGGCCGTCGTTTTACAACGTCGTGA
CTGGGAAAGGCGCGCCAGATCGCAAAAAACAGTACATACAGAAGGAGACATGAACTAAAAGTTTTGTTACTTTATAGAAGAAATTTTGAGTTT
TTGTTTTTTTTTAATAAATAAATAAACATAAATAAATTGTTTGTTGAATTTATTATTAGTATGTAAGTGTAAATATAATAAAACTTAATATCTATTC
AAATTAATAAATAAACCTCGATATACAGACCGATAAAACACATGCGTCAATTTTACGCATGATTATCTTTAACGTACGTCACAATATGATTATCT
TTCTAGGGTTAACAACTATGGATATAAAATAGGTACTAATCAAAATAGTGAGGAGGATATATTTGAATACATACGAACAAATTAATAAAGTGA
AAAAAATACTTCGGAAACATTTAAAAAATAACCTTATTGGTACTTACATGTTTGGATCAGGAGTTGAGAGTGGACTAAAACCAAATAGTGATCT
TGACTTTTTAGTCGTCGTATCTGAACCATTGACAGATCAAAGTAAAGAAATACTTATACAAAAAATTAGACCTATTTCAAAAAAAAATAGGAGAT
AAAAGCAACTTACGATATATTGAATTAACAATTATTATTCAGCAAGAAATGGTACCGTGGAATCATCCTCCCAAACAAGAATTTATTTATGGAG
AATGGTTACAAGAGCTTTATGAACAAGGATACATTCCTCAGAAGGAATTAAATTCAGATTTAACCATAATGCTTTACCAAGCAAAACGAAAAA
ATAAAAGAATATACGGAAATTATGACTTAGAGGAATTACTACCTGATATTCCATTTTCTGATGTGAGAAGAGCCATTATGGATTCGTCAGAGGA
ATTAATAGATAATTATCAGGATGATGAAACCAACTCTATATTAACTTTATGCCGTATGATTTTAACTATGGACACGGGTAAAATCATACCAAAA
GATATTGCGGGAAATGCAGTGGCTGAATCTTCTCCATTAGAACATAGGGAGAGAATTTTGTTAGCAGTTCGTAGTTATCTTGGAGAGAATATTGA

ATGGACTAATGAAAATGTAAATTTAACTATAAACTATTTAAATAACAGATTAAAAAAATTATAATGATAGACGCCGGGATAACTCCGGCGTTTT
T**<u>TTTGTTCAAAAAAAAGCCCGCTC</u>**ATTAGGCGGGCTGGGTTATATTCCCCAGAACATCAGGTTAATGGCGTTTTTGATGTCATTTTCGCGGTGGC
TGAGATCAGCCACTTCTTCCCCGATAACGGAGACCGGCACACTGGCCATATCGGTGGTCATCATGCGCCAGCTTTCATCCCCGATATGCACCAC
CGGGTAAAGTTCACGGGAGACTTTATCTGACAGCAGACGTGCACTGGCCAGGGGGATCACCATCCGTCGCCCGGGCGTGTCAATAATATCACTC
TGTACATCCACAAACAGACGATAACGGCTCTCTCTTTTATAGGTGTAAACCTTAAACTGCATAATCTGACCTCCTGGTTATGTGTGGGAGGGCTA
ACCATGGATCCATGGTTACCAATGCTTAATCAGTGAGGCACCTATCTCAGCGATCTGTCTATTTCGTTCATCCATAGTTGCCTGACTCCCCGTCGT
GTAGATAACTACGATACGGGAGGGCTTACCATCTGGCCCCAGTGCTGCAATGATACCGCGAGACCCACGCTCACCGGCTCCAGATTTATCAGCA
ATAAACCAGCCAGCCGGAAGGGCCGAGCGCAGAAGTGGTCCTGCAACTTTATCCGCCTCCATCCAGTCTATTAATTGTTGCCGGGAAGCTAGAG
TAAGTAGTTCGCCAGTTAATAGTTTGCGCAACGTTGTTGCCATTGCTGCAGGCATCGTGGTGTCACGCTCGTCGTTTGGTATGGCTTCATTCAGCT
CCGGTTCCCAACGATCAAGGCGAGTTACATGATCCCCCATGTTGTGCAAAAAAGCGGTTAGCTCCTTCGGTCCTCCGATCGTTGTCAGAAGTAA
GTTGGCCGCAGTGTTATCACTCATGGTTATGGCAGCACTGCATAATTCTCTTACTGTCATGCCATCCGTAAGATGCTTTTCTGTGACTGGTGAGTA
CTCAACCAAGTCATTCTGAGAATAGTGTATGCGGCGACCGAGTTGCTCTTGCCCGGCGTCAACACGGGATAATACCGCGCCACATAGCAGAACT
TTAAAAGTGCTCATCATTGGAAAACGTTCTTCGGGGCGAAAACTCTCAAGGATCTTACCGCTGTTGAGATCCAGTTCGATGTAACCCACTCGTGC
ACCCAACTGATCTTCAGCATCTTTTACTTTCACCAGCGTTTCTGGGTGAGCAAAAACAGGAAGGCAAAATGCCGCAAAAAAGGGAATAAGGGC
GACACGGAAATGTTGAATACTCATACTCTTCCTTTTTCAATATTATTGAAGCATTTATCAGGGTTATTGTCTCATGAGCGGATACATATTTGAATG
TATTTAGAAAAATAAACAAA

B. pR6K-(IP)-ccdB-Amp-(IP)

GCTACCTTAAGAGAGTGTCAGC***CGTTAAGTGTTCCTGTGTCACTC***AAAATTGCTTTGAGAGGCTCTAAGGGCTTCTCAGTGCGTTACATCCCTGGCTTGTT
GTCCACAACCGTTAAACCTTAAAAGCTTTAAAAGCCTTATATATTCTTTTTTTTCTTATAAAACTTAAAACCTTAGAGGCTATTTAAGTTGCTGATTTATAT
TAATTTTATTGTTCAAACATGAGAGCTTAGTACGTGAAACATGAGAGCTTAGTACGTTAGCCATGAGAGCTTAGTACGTTAGCCATGAGGGTTTAGTTCG
TTAAACATGAGAGCTTAGTACGTTAAACATGAGAGCTTAGTACGTGAAACATGAGAGCTTAGTACGTACTATCAACAGGTTGAACTGCTGATCTTCAGA
TCCTCTACGCCGGACGCATCGTGGCCGTTTTCCGCTGCATAACCCTGCTTCGGGGTCATTATAGCGATTTTTTCGGTATATCCATCCTTTTTCGCACGATAT
ACAGGATTTTGCCAAAGGGTTCGTGTAGACTTTCCTTGGTGTATCCAACGGCGTCAGCCGGGCAGGATAGGTGAAGTAGGCCCACCCGCGAGCGGGTGT
TCCTTCTTCACTGTCCCTTATTCGCACCTTAATTAATTCACTGGCCGTCGTTTTACAACGTCGTGACTGGGAAAGGCGCGCCAGATCGCAAAAAACAGTA
CATACAGAAGGAGACATGAACTAAAAGTTTTGTTACTTTATAGAAGAAATTTTGAGTTTTTGTTTTTTTTTAATAAATAAATAAACATAAATAAATTGTTT
GTTGAATTTATTATTAGTATGTAAGTGTAAATATAATAAAACTTAATATCTATTCAAATTAATAAATAAACCTCGATATACAGACCGATAAAACACATGC
GTCAATTTTACGCATGATTATCTTTAACGTACGTCACAATATGATTATCTTTCTAGGGTTAACAACTATGGATATAAAATAGGTACTAATCAAAATAGTG
AGGAGGATATATTTGAATACATACGAACAAATTAATAAAGTGAAAAAAATACTTCGGAAACATTTAAAAAATAACCTTATTGGTACTTACATGTTTGGA
TCAGGAGTTGAGAGTGGACTAAAACCAAATAGTGATCTTGACTTTTTAGTCGTCGTATCTGAACCATTGACAGATCAAAGTAAAGAAATACTTATACAA

AAAATTAGACCTATTTCAAAAAAAATAGGAGATAAAAGCAACTTACGATATATTGAATTAACAATTATTATTCAGCAAGAAATGGTACCGTGGAATCA
TCCTCCCAAACAAGAATTTATTTATGGAGAATGGTTACAAGAGCTTTATGAACAAGGATACATTCCTCAGAAGGAATTAAATTCAGATTTAACCATAAT
GCTTTACCAAGCAAAACGAAAAAATAAAAGAATATACGGAAATTATGACTTAGAGGAATTACTACCTGATATTCCATTTTCTGATGTGAGAAGAGCCAT
TATGGATTCGTCAGAGGAATTAATAGATAATTATCAGGATGATGAAACCAACTCTATATTAACTTTATGCCGTATGATTTTAACTATGGACACGGGTAAA
ATCATACCAAAAGATATTGCGGGAAATGCAGTGGCTGAATCTTCTCCATTAGAACATAGGGAGAGAATTTTGTTAGCAGTTCGTAGTTATCTTGGAGAG
AATATTGAATGGACTAATGAAAATGTAAATTTAACTATAAACTATTTAAATAACAGATTAAAAAAATTATAATGAT**AGACGCCGGGATAACTCC**GGCG
TTTTTTTTAACCCTAGAACTCTCTTAAGGTAGCTTTGTTCAAAAAAAAGCCCGCTCATTAGGCGGGCTGGGTTATATTCCCCAGAACATCAGGTTAATGG
CGTTTTTGATGTCATTTTCGCGGTGGCTGAGATCAGCCACTTCTTCCCCGATAACGGAGACCGGCACACTGGCCATATCGGTGGTCATCATGCGCCAGCT
TTCATCCCCGATATGCACCACCGGGTAAAGTTCACGGGAGACTTTATCTGACAGCAGACGTGCACTGGCCAGGGGGATCACCATCCGTCGCCCGGGCGT
GTCAATAATATCACTCTGTACATCCACAAACAGACGATAACGGCTCTCTCTTTTATAGGTGTAAACCTTAAACTGCATAATCTGACCTCCTGGTTATGTGT
GGGAGGGCTAACCATGGATCCATGGTTACCAATGCTTAATCAGTGAGGCACCTATCTCAGCGATCTGTCTATTTCGTTCATCCATAGTTGCCTGACTCCC
CGTCGTGTAGATAACTACGATACGGGAGGGCTTACCATCTGGCCCCAGTGCTGCAATGATACCGCGAGACCCACGCTCACCGGCTCCAGATTTATCAGC
AATAAACCAGCCAGCCGGAAGGGCCGAGCGCAGAAGTGGTCCTGCAACTTTATCCGCCTCCATCCAGTCTATTAATTGTTGCCGGGAAGCTAGAGTAA
GTAGTTCGCCAGTTAATAGTTTGCGCAACGTTGTTGCCATTGCTGCAGGCATCGTGGTGTCACGCTCGTCGTTTGGTATGGCTTCATTCAGCTCCGGTTCC
CAACGATCAAGGCGAGTTACATGATCCCCCATGTTGTGCAAAAAAGCGGTTAGCTCCTTCGGTCCTCCGATCGTTGTCAGAAGTAAGTTGGCCGCAGTG
TTATCACTCATGGTTATGGCAGCACTGCATAATTCTCTTACTGTCATGCCATCCGTAAGATGCTTTTCTGTGACTGGTGAGTACTCAACCAAGTCATTCTG
AGAATAGTGTATGCGGCGACCGAGTTGCTCTTGCCCGGCGTCAACACGGGATAATACCGCGCCACATAGCAGAACTTTAAAAGTGCTCATCATTGGAA
AACGTTCTTCGGGGCGAAAACTCTCAAGGATCTTACCGCTGTTGAGATCCAGTTCGATGTAACCCACTCGTGCACCCAACTGATCTTCAGCATCTTTTAC
TTTCACCAGCGTTTCTGGGTGAGCAAAAACAGGAAGGCAAAATGCCGCAAAAAAGGGAATAAGGGCGACACGGAAATGTTGAATACTCATACTCTTCC
TTTTTCAATATTATTGAAGCATTTATCAGGGTTATTGTCTCATGAGCGGATACATATTTGAATGTATTTAGAAAAATAAACAAA

C. pR6K-(AB)-ccdB-Amp-(AB)

CCTCGAGG***TGTCAGCCGTTAAGTGTTCCT***GTGTCACTCAAAATTGCTTTGAGAGGCTCTAAGGGCTTCTCAGTGCGTTACATCCCTGGCTTGTTGT
CCACAACCGTTAAACCTTAAAAGCTTTAAAAGCCTTATATATTCTTTTTTTTCTTATAAAACTTAAAACCTTAGAGGCTATTTAAGTTGCTGATTT
ATATTAATTTTATTGTTCAAACATGAGAGCTTAGTACGTGAAACATGAGAGCTTAGTACGTTAGCCATGAGAGCTTAGTACGTTAGCCATGAGG
GTTTAGTTCGTTAAACATGAGAGCTTAGTACGTTAAACATGAGAGCTTAGTACGTGAAACATGAGAGCTTAGTACGTACTATCAACAGGTTGAA
CTGCTGATCTTCAGATCCTCTACGCCGGACGCATCGTGGCCGTTTTCCGCTGCATAACCCTGCTTCGGGGTCATTATAGCGATTTTTTCGGTATAT
CCATCCTTTTTCGCACGATATACAGGATTTTGCCAAAGGGTTCGTGTAGACTTTCCTTGGTGTATCCAACGGCGTCAGCCGGGCAGGATAGGTGA
AGTAGGCCCACCCGCGAGCGGGTGTTCCTTCTTCACTGTCCCTTATTCGCACCTTAATTAATTCACTGGCCGTCGTTTTACAACGTCGTGACTGGG
AAAGGCGCGCCAGATCGCAAAAAACAGTACATACAGAAGGAGACATGAACTAAAAGTTTTGTTACTTTATAGAAGAAATTTTGAGTTTTTGTTT
TTTTTTAATAAATAAATAAACATAAATAAATTGTTTGTTGAATTTATTATTAGTATGTAAGTGTAAATATAATAAAACTTAATATCTATTCAAATT
AATAAATAAACCTCGATATACAGACCGATAAAACACATGCGTCAATTTTACGCATGATTATCTTTAACGTACGTCACAATATGATTATCTTTCTA
GGGTTAACAACTATGGATATAAAATAGGTACTAATCAAAATAGTGAGGAGGATATATTTGAATACATACGAACAAATTAATAAAGTGAAAAAA
ATACTTCGGAAACATTTAAAAAATAACCTTATTGGTACTTACATGTTTGGATCAGGAGTTGAGAGTGGACTAAAACCAAATAGTGATCTTGACTT
TTTAGTCGTCGTATCTGAACCATTGACAGATCAAAGTAAAGAAATACTTATACAAAAAATTAGACCTATTTCAAAAAAAAATAGGAGATAAAAG
CAACTTACGATATATTGAATTAACAATTATTATTCAGCAAGAAATGGTACCGTGGAATCATCCTCCCAAACAAGAATTTATTTATGGAGAATGG
TTACAAGAGCTTTATGAACAAGGATACATTCCTCAGAAGGAATTAAATTCAGATTTAACCATAATGCTTTACCAAGCAAAACGAAAAAATAAA
AGAATATACGGAAATTATGACTTAGAGGAATTACTACCTGATATTCCATTTTCTGATGTGAGAAGAGCCATTATGGATTCGTCAGAGGAATTAAT
AGATAATTATCAGGATGATGAAACCAACTCTATATTAACTTTATGCCGTATGATTTTAACTATGGACACGGGTAAAATCATACCAAAAGATATT
GCGGGAAATGCAGTGGCTGAATCTTCTCCATTAGAACATAGGGAGAGAATTTTGTTAGCAGTTCGTAGTTATCTTGGAGAGAATATTGAATGGA
CTAATGAAAATGTAAATTTAACTATAAACTATTTAAATAACAGATTAAAAAAATTATAATGATAGACGCCGGGATAACTCCGGCGTTTTTT**TTC**
CTCGAGGTCATAGCCCATATATGGAGTTTGTTTATTTTTCTAAATACATTCAAATATGTATCCGCTCATGAGACAATAACCCTGATAAATGCTTC
AATAATATTGAAAAAGGAAGAGTATGAGTATTCAACATTTCCGTGTCGCCCTTATTCCCTTTTTTGCGGCATTTTGCCTTCCTGTTTTTGCTCACCC
AGAAACGCTGGTGAAAGTAAAAGATGCTGAAGATCAGTTGGGTGCACGAGTGGGTTACATCGAACTGGATCTCAACAGCGGTAAGATCCTTGA
GAGTTTTCGCCCCGAAGAACGTTTTCCAATGATGAGCACTTTTAAAGTTCTGCTATGTGGCGCGGTATTATCCCGTGTTGACGCCGGGCAAGAGC
AACTCGGTCGCCGCATACACTATTCTCAGAATGACTTGGTTGAGTACTCACCAGTCACAGAAAAGCATCTTACGGATGGCATGACAGTAAGAGA
ATTATGCAGTGCTGCCATAACCATGAGTGATAACACTGCGGCCAACTTACTTCTGACAACGATCGGAGGACCGAAGGAGCTAACCGCTTTTTTG
CACAACATGGGGGATCATGTAACTCGCCTTGATCGTTGGGAACCGGAGCTGAATGAAGCCATACCAAACGACGAGCGTGACACCACGATGCCT
GCAGCAATGGCAACAACGTTGCGCAAACTATTAACTGGCGAACTACTTACTCTAGCTTCCCGGCAACAATTAATAGACTGGATGGAGGCGGAT
AAAGTTGCAGGACCACTTCTGCGCTCGGCCCTTCCGGCTGGCTGGTTTATTGCTGATAAATCTGGAGCCGGTGAGCGTGGGTCTCGCGGTATCAT

TGCAGCACTGGGGCCAGATGGTAAGCCCTCCCGTATCGTAGTTATCTACACGACGGGGAGTCAGGCAACTATGGATGAACGAAATAGACAGAT
CGCTGAGATAGGTGCCTCACTGATTAAGCATTGGTAACCATGGATCCATGGTTAGCCCTCCCACACATAACCAGGAGGTCAGATTATGCAGTTT
AAGGTTTACACCTATAAAAGAGAGAGCCGTTATCGTCTGTTTGTGGATGTACAGAGTGATATTATTGACACGCCCGGGCGACGGATGGTGATCC
CCCTGGCCAGTGCACGTCTGCTGTCAGATAAAGTCTCCCGTGAACTTTACCCGGTGGTGCATATCGGGGATGAAAGCTGGCGCATGATGACCAC
CGATATGGCCAGTGTGCCGGTCTCCGTTATCGGGGAAGAAGTGGCTGATCTCAGCCACCGCGAAAATGACATCAAAAACGCCATTAACCTGAT
GTTCTGGGGAATATAACCCAGCCCGCCTAATGAGCGGGCTTTTTTTTGAACAAAGTTAACAACAACAATTGCATTC

Supplementary Methods: Generation of the new selection marker containing plasmids

To introduced the I-PpoI and AbsI recognition site into the previously described ccdB counter-selection marker (SM) plasmid [1], plasmid pR6K-hyg-spect-PBs [2] was digest with SphI and BstAPI, then co-electroporated with the respective restriction enzyme site containing PCR product (see the table below) into arabinose induced competent *E.coli* GBdir-pir116-gyrA462. After recovery at 37 °C for 1 h, the bacterial were streak on LB-Agar plate containing spectinomycin (60 ug/ml) and Ampicillin (100 µg/ml) for selection overnight. The colonies were picked for further culture, then checked via restriction digest, sequence confirmation and function test.

Oligonucleotides used to generate the new selection marker containing plasmids:

Name	Sequence	Note /application
RS-ccdB-HAF		PCR to generate the pR6K-(IP)-ccdB-Amp plasmid, the I-PpoI site is in uppercase letters.
RS-IP-ccdb-HAR	agcaattttgagtgacacaggaacacttaacggctgacaCTCTCTTAAGGTAGCtttgtttatttttctaaatac	
RS-IP-SM-fwd	agacgccgggataactccggcgtttttttaaccctagaaCTCTCTTAAGGTAGCtttgttcaaaaaaaagcccgct	PCR to generate the pR6K-(IP)-ccdB-Amp-(IP) plasmid, the I-PpoI sites are in uppercase letters.
RS-IP-ccdb-HAR	agcaattttgagtgacacaggaacacttaacggctgacaCTCTCTTAAGGTAGCtttgtttatttttctaaatac	
AbsI-SM-fwd	cagattaaaaaaattataatgatagacgccgggataactccggcgttttttttCCTCGAGGtcatagcccatatatggagt	PCR to generate the pR6K-(AB)-ccdB-Amp-(AB) plasmid, the AbsI sites are in uppercase letters.
AbsI-SM-rev	tagagcctctcaaagcaattttgagtgacacaggaacacttaacggctgacaCCTCGAGGgaatgcaattgttgttgttaac	

REFERENCE

1. Wang, H.; Bian, X.; Xia, L.; Ding, X.; Muller, R.; Zhang, Y.; Fu, J.; Stewart, A.F. Improved seamless mutagenesis by recombineering using ccdB for counterselection. *Nucleic Acids Res* **2014**, *42*, e37, doi:10.1093/nar/gkt1339.
2. Rostovskaya, M.; Fu, J.; Obst, M.; Baer, I.; Weidlich, S.; Wang, H.; Smith, A.J.; Anastassiadis, K.; Stewart, A.F. Transposon-mediated BAC transgenesis in human ES cells. *Nucleic Acids Res* **2012**, *40*, e150, doi:10.1093/nar/gks643.
